# Ginsenoside Rb3 Alleviates the Toxic Effect of Cisplatin on the Kidney during Its Treatment to Oral Cancer via TGF-*β*-Mediated Mitochondrial Apoptosis

**DOI:** 10.1155/2021/6640714

**Published:** 2021-01-16

**Authors:** Wen-Jie Wu, Yu-Fang Tang, Shuang Dong, Jie Zhang

**Affiliations:** Department of Oral and Maxillofacial Surgery, Peking University School and Hospital of Stomatology, Beijing, China

## Abstract

**Objective:**

The research aimed to confirm the role of the transforming growth factor-*β* (TGF-*β*) in cisplatin- (CPT-) evoked kidney toxicity and elucidate the mechanism that ginsenoside Rb3 (Rb3) could alleviate the kidney toxicity by CPT during its treatment to oral cancer via TGF-*β*-mediated mitochondrial apoptosis.

**Methods:**

The model of xenograft nude mice bearing oral carcinoma cells ACC83 was established and treated with CPT and/or Rb3, respectively. Bodyweights of the treated mice were weighed, and the kidney tissues were collected; following, the histopathology and the expression of TGF-*β* were examined using H&E staining and immunohistochemistry. Afterward, the renal cells GP-293 were treated with CPT and/or Rb3. The expression and phosphoration of TGF-*β*, Smad2, Smad3, Bcl-2, and Bax in GP-293 cells were detected by Western blotting. The cellular apoptosis and mitochondrial membrane potential were analyzed using flow cytometry.

**Results:**

The xenograft nude mice exposure to CPT presented the bodyweight loss, necrotic areas, and the increased expression of TGF in kidney tissue, and Rb3 pretreatment relieved these changes evoked by CPT. In GP-293 cells, CPT administration induced the phosphorylation of Smad2 and Smad3, and Rb3 pretreatment suppressed the induced phosphorylation by CPT. Besides, flow cytometry analysis showed that Rb3 inhibited the CPT-evoked cellular apoptosis ratio and mitochondrial membrane depolarization. The Western blotting test indicated that Rb3 alleviated the cleavage of PARP, caspase 3, caspase 8, and caspase 9, the induction of Bax expression, and inhibition of Bcl-2 expression. Additionally, after treating with the TGF inhibitor of disitertide, Rb3 exhibited no alleviation effects on CPT-evoked cellular apoptosis ratio, inhibition of Bax expression, and induction of Bcl-2 expression in GP-293 cells.

**Conclusion:**

Rb3 could alleviate CPT-evoked toxic effects on kidney cells during its treatment to oral cancer via TGF-*β*-mediated mitochondrial apoptosis.

## 1. Introduction

Oral cancer, occurring in the oral cavity and lip, is the sixth most common carcinoma and accounts for approximately 300,000 new cases around the earth [1]. The lack of early diagnosis strategy to oral cancer delayed the treatment and led to the dismal survival rates and mortality in oral cancer [2–5]. According to the statistics, there are 145,000 patients of oral cancer who died in 2012 [1]. Oral cancer has caused an alarming mental and health burden in human [6, 7]. Although there are plentiful of therapy methods for oral cancer in clinic and theory, the effective strategy for cancer treatment remains a challenge [8, 9]. Currently, chemotherapy is still one of the usual therapies to various cancers including oral cancer [10], and cisplatin (CPT) is an essential chemotherapeutical agent wildly applied to oral cancer for years [11, 12]. However, CPT could kill almost all of the contacted cells of normal and cancer cells in the body, which lead to the toxicity to normal tissues such as the heart, kidney, and liver [13]. The lack of selectivity to cells restricted the clinic application of CPT in treating against oral cancer [14]. Excitingly, recent research studies have shown that several traditional Chinese herb extractions and their components could suppress CPT-trigged liver impairment, kidney injury, and myocardial damage [15, 16]. Among these active herb extractions and components, it was reported that Rb3 could alleviate CPT-evoked kidney injury in the cancer model [17]. However, the potential effect and mechanism of Rb3 on the kidney toxicity during CTP treating against oral cancer remain obscure. Therefore, it is urgent to elucidate the kidney protective role of Rb3 in the process of CPT-treating oral cancer for Rb3's medicinal development and the clinic treatment of oral cancer.

TGF-*β* pathway is considered as an essential process of signal cascade response to the transforming growth to mediate cell proliferation, apoptosis, and differentiation [18]. It exerts a variety of pathological and physiological function in the tumor progression, wound healing, immune response, and so on [19, 20]. Recently, several emerging shreds of evidences have shown that TGF-*β* could serve as a tissue repair mediator to benefit or exacerbate the tissue damage in the brain, liver, and other tissues via mitochondrial apoptosis [21, 22]. Jin et al. reported that TGF-*β* contributed to LRG1-induced ischemia/reperfusion injury via apoptosis [23]. Another study by Wang et al. also indicated that TGF-*β* participated in the miRNA-590-5p-regulated chondrocyte apoptosis in response to the mechanical pressure injury [24]. Mitochondrial apoptosis is exceptionally essential to maintain the stabilization of homeostasis in the body, which could clean up the unwanted and abnormal cells according to a serious of programmed signal instruction of apoptosis factors. However, the disorder and despitefully employed apoptosis would result in normal cell death and tissue injury [25]. In the treatment of cancer by CPT, enormous shreds of evidence confirmed that CPT induced the cell apoptosis and tissue injury in the normal kidney, liver, lung, and other tissues and eventually caused the severe toxicity effect [26]. This information implies that CPT-evoked kidney toxicity during the treatment of oral cancer may be associated to the TGF-*β*-induced mitochondrial apoptosis.

In the present study, we aimed to confirm the role of TGF-*β* in the CPT-evoked kidney toxicity during the treatment of oral cancer and elucidate the underlying mechanism that Rb3 alleviates kidney toxicity by CPT via TGF-*β*-mediated mitochondrial apoptosis. First, the xenograft nude mice model bearing oral carcinoma cells ACC83 was constructed. The kidney toxicity by CPT, the protective effect of Rb3, and the TGF-*β* expression in kidney tissue were evaluated. Second, the human renal epithelial GP-293 cells were employed to determine the effect of Rb3 and CPT on the TGF-*β* signal pathway and mitochondrial apoptosis. Finally, the role of the TGF-*β* signal pathway on Rb3 suppressing mitochondrial apoptosis evoked by CPT in GP-293 cells was studied.

## 2. Materials and Methods

### 2.1. Chemicals and Reagents

The standards of ginsenoside Rb3 and cisplatin was obtained from Aladdin (China). Hematoxylin and eosin were purchased from Nanjing Jiancheng (China). The primary antibody of TGF-*β*, Smad2, Smad3, p-Smad2, p-Smad3, Bcl-2, Bax, cleaved PARP, cleaved caspase 3, cleaved caspase 8, cleaved caspase 9, and *β*-actin were supplied from Abcam (USA). Annexin V-FITC apoptosis kit was purchased from BD (USA). JC-1 dye was obtained from Solarbio (China). Caspase 3, caspase 8, and caspase 9 colorimetric assay kits were purchased from BioVision (USA).

### 2.2. Establishing the Xenograft Nude Mice Model Bearing Oral Carcinoma Cells ACC83

The SPF male and 6 weeks aged nude mice were purchased from the Xiamen University Laboratory Animal Center (Xiamen, China). All mice were housed according to genotype and sex, five per cage and raised at the atmosphere of 22–26°C on a 12-hour light-dark cycle with continuous access to food and water. After adapting the environment for five days, the mice were subcutaneously implanted with the precultured oral carcinoma cells ACC83 (approximately 5 × 10^6^ cells in 100 *μ*L PBS per mice). One week later, when the implanted ACC83 cells began to display a little tumor swell with different sizes in the subcutaneous of mice, we selected the mice bearing tumor of uniform size as mung beans and randomly divided into four groups (*n* = 6). The selected mice were maintained for another one week and treated with CPT and/or Rb3 to evaluate the toxic effect.

### 2.3. Animal Experiments Design and Treatment

The nude mice bearing oral tumor were divided into four groups: control, CPT, CPT +  low Rb3, and CPT + high Rb3 groups. The control group was administrated with saline once daily for continuous 28 days. The CPT group was intraperitoneally injected with 10 mg/kg cisplatin every other day for 28 days. The CPT + low Rb3 and CPT + high Rb3 groups were administrated with 10 mg/kg and 20 mg/kg ginsenoside Rb3, respectively, once daily, and the same cisplatin injection for the CPT group for 28 days. Bodyweight of mice was measured once weekly. At the end of the treatment on 28^th^ day, all mice were euthanized, and the kidneys were immediately dissected out and fixed with 4% paraformaldehyde for histopathological analysis.

### 2.4. H&E Staining for Histopathology

After euthanizing, the kidneys from all groups of nude mice were collected. The left kidneys were washed with precooled phosphate buffer saline (PBS) and stored in an ultralow temperature freezer of −80°C for the following Western blotting experiment. The right kidneys were cut into the squares (0.7 cm × 0.7 cm) and fixed with 4% paraformaldehyde at 4°C for more than 24 hours. The fixed squares of kidneys were embedded in paraffin, sliced in 3 *μ*m thickness, and then adhered to slides. The sliced thin kidney samples were dewaxed via continuously placing in the solution of xylene I for 10 min, xylene II for 10 min, anhydrous alcohol I for 5 min, anhydrous alcohol II for 5 min, 95% alcohol for 5 min, 90% alcohol for 5 min, 80% alcohol for 5 min, and 70% alcohol for 5 min, respectively, and then washed with distilled water. The dewaxed slides were further stained with hematoxylin for 5 min and eosin for 3 min. At last, the stained kidney tissues were dehydrated in the solution of 95% alcohol, anhydrous alcohol, and xylene in tune and coverslipped with neutral resins for detecting and imaging using a fluorescence microscope (Leica, Germany).

### 2.5. Immunohistochemistry for the Expression of TGF-*β*

The above dewaxed slices were put into the boiled solution of citrate for antigen retrieval and then blocked in 10% goat serum at room temperature for 1 hour. The blocking slices were added 50 *μ*L diluted solution of a primary antibody of TGF-*β* (1 : 500 dilution) and incubated at 37°C for 1 hour, washed with PBS for three times followed by the secondary antibody for another 1 hour. After washing with PBS for three times, the slices were colored with 50 *μ*L amount of the fresh chromogenic reagent of DAB and stained with hematoxylin for 30 seconds. The running water washed the slices for 20 minutes to cease the staining. Finally, the stained kidney tissues were dehydrated in the solution of 95% alcohol I for 3 seconds, 95% alcohol II for 3 seconds, anhydrous alcohol for 5 minutes, xylene-alcohol (1 : 1) for 5 minutes, xylene I for 2 minutes, and xylene II for 2 minutes in tune and coverslipped with neutral resins for detecting and imaging using a fluorescence microscope (Leica, Germany).

### 2.6. Cell Culture and Treatments

The oral carcinoma ACC83 cells and normal renal epithelial cells GP-293 were maintained in Dulbecco's modified Eagle's medium (DMEM) containing 10% FBS in a 5% CO_2_ atmosphere at 37°C. When the amounts of ACC cells in the logarithmic phase reached to 12 of 10 cm cell culture plates with 90% confluence, they were digested with 0.25% trypsin solution and resuspended into 5 mL EP tubes with PBS according to 200 *μ*L PBS per 10 cm cell culture plate; following, the resuspended cells were subcutaneously injected into nude mice at 100 *μ*L for one mouse to construct the oral tumor mice model. When GP-293 cells in the logarithmic phase were about 85% confluence, they were seeded into 6 wells cultured plate and treated with CPT and/or Rb3 as necessary. And then, the cells were collected and detected by the following experiments.

### 2.7. Flow Cytometry of Dual-Staining of FITC-Annexin V/PI for the Cellular Apoptosis

The GP-293 cells treated by CTP and/or Rb3 were digested into single cells with 0.25% trypsin and transferred into 1.5 mL EP tubes. The digested cells were washed with precold PBS and centrifuged at 1500 rpm and 4°C for 5 minutes. The precipitate of cells was resuspended with 300 *μ*L 1 × binding buffer; following, 5 *μ*L Annexin V-FITC was added to incubate at room temperature away from light for 15 minutes, and then, 5 *μ*L of PI was added to stain. At last, the stained cells were supplied with another 200 *μ*L 1 × binding buffer and detected by the flow cytometry (Bechman, USA).

### 2.8. Western Blotting for Detecting the Expression of the Protein

The GP-293 cells treated by CTP and/or Rb3 were harvested and lysed with RIPA buffer. The total protein concentration of cell lysates was detected using BCA kit and then was denatured with loading buffer under the condition of boiling. 30 *μ*g of the total denatured proteins were loaded and subjected to the sodium dodecyl sulphate‐polyacrylamide gel electrophoresis (SDS-PAGE) for protein separation. The separated proteins were transferred to PVDF membrane. After blocking with 5% skim milk in Tris buffered saline with Tween 20 (TBST) for 1 hour, the PVDF membranes were incubated with the primary antibodies against TGF-*β*, Smad2, Smad3, p-Smad2, p-Smad3, Bcl-2, Bax, cleaved PARP, cleaved caspase 3, cleaved caspase 8, and cleaved caspase 9 at 4°C overnight, and the antibody of *β*-actin was employed as an internal reference. The PVDF membranes were washed with TBST three times and further incubated with the corresponding secondary antibody at room temperature for 2 hours. Finally, the corresponding target on the PVDF membrane was probed using ECL reagent.

### 2.9. Flow Cytometry of JC-1 Staining for the Mitochondrial Potential

Following the treatment of CPT and/or Rb3 in GP-293 cells, the medium was discarded, and the cells were washed using PBS, and fresh culture medium (1 mL) was added. Following, 1 mL of JC-1 working solution was added into the cells to incubate at 37°C for additional 20 minutes. After incubation, the cells were washed twice with the precold JC-1 staining buffer and digested with 0.25% trypsin. The digested cells were used for flow cytometry analysis.

### 2.10. Statistical Analysis

All of the experiments data were presented as the mean ± standard deviation (S.D.). The statistical differences among the groups were compared using one-way ANOVA by SPSS of version 19.0 (SPSS, USA). *p* < 0.05 was considered to be statistically significant. The asterisk (^*∗*^) represented the comparison with the control group, and the pound sign (^#^) was for the comparison with the CPT group.

## 3. Results

### 3.1. The Protective Effects of Rb3 on CPT-Evoked Kidney Toxicity in Xenograft Nude Mice

**Figure 1:**
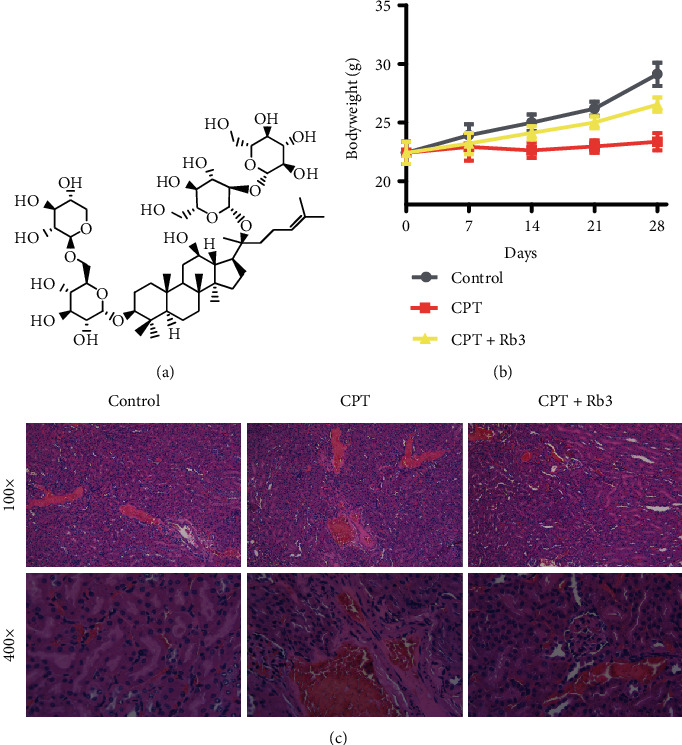
Rb3 protected against CPT-evoked kidney toxicity in xenograft nude mice. (a) The structure of ginsenoside Rb3. (b) The curves of bodyweight of mice. (c) H&E staining of kidney tissue.

Bodyweight and histopathology are standard methods for the evaluation of medicinal toxicity. In the control group, the bodyweight of xenograft nude mice bearing oral carcinoma cells ACC83 had a linear increase from 0 to 28 days (Figure 1(b)). The mice injected with CPT displayed a horizontal linearity in bodyweight which was evident beneath compared with that of the control group, demonstrating that CPT caused the bodyweight loss of mice. Expectedly, ginsenoside Rb3 (the structure shown in Figure 1(a)) administration by gavage at 20 mg/kg dosage in CPT + Rb3 groups alleviated the loss of bodyweight caused by CPT as determined by the bodyweight curve close to that of the control group. Furthermore, histopathology analysis (Figure 1(c)) showed that numerous necrotic areas were found in the kidney tissue of the CPT group rather than that of the control group. Meanwhile, ginsenoside Rb3 pretreatment inhibited the necrosis of kidney tissue caused by CPT. These results indicated that ginsenoside Rb3 could protect against the CPT-evoked kidney toxicity in xenograft nude mice bearing an oral tumor.

### 3.2. The Effects of Rb3 on the Expression Level of TGF-*β* in Kidney Tissue of Xenograft Nude Mice

**Figure 2:**
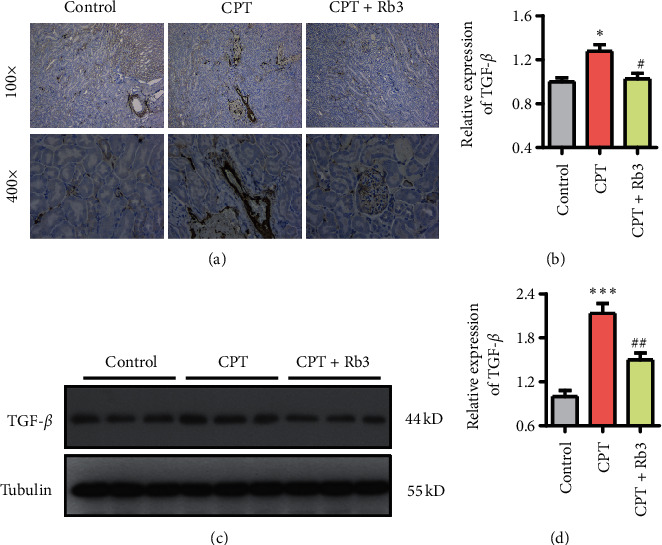
Rb3 suppressed the TGF-*β* expression evoked by CPT in kidney tissue of xenograft nude mice. (a) Immunohistochemistry for TGF-*β* expression in kidney tissue. (b) Quantitative analysis of TGF-*β* expression by immunohistochemistry. (c) Western blotting for TGF-*β* expression in kidney tissue. (d) Quantitative analysis of TGF-*β* expression by Western blotting. ^*∗*^*p* < 0.05 and ^*∗∗*^*p* < 0.01 compared to the control group; ^#^*p* < 0.05 compared to the CPT group.

The level of TGF-*β* expression was examined with immunohistochemical staining and Western blotting. As seen in the representative images of immunohistochemical (Figure 2(a)), kidney tissue of mice in the control group presented a normal cellular morphology and TGF-*β* expression. In contrast, CPT injection promoted the induction of deformed cellular morphology and TGF-*β* expression compared with these of the control group (^*∗*^*p* < 0.01, Figure 2(b)). Conversely, Rb3 administration at 20 mg/kg dose suppressed CPT-induced TGF-*β* expression and repaired the deformed cellular morphology in kidney tissue, and its levels were similar with these in the control group (Figures 2(a) and 2(b)). Additionally, Western blotting assay revealed the similar changing trend of TGF-*β* expression with data obtained by immunohistochemical staining, which was that CPT induced the TGF-*β* expression and Rb3 suppressed the induction of TGF-*β* expression by CTP in kidney tissues of xenograft nude mice bearing oral tumor.

### 3.3. The Effects of Rb3 on the TGF-*β* Signal Pathway in GP-293 Cells

**Figure 3:**
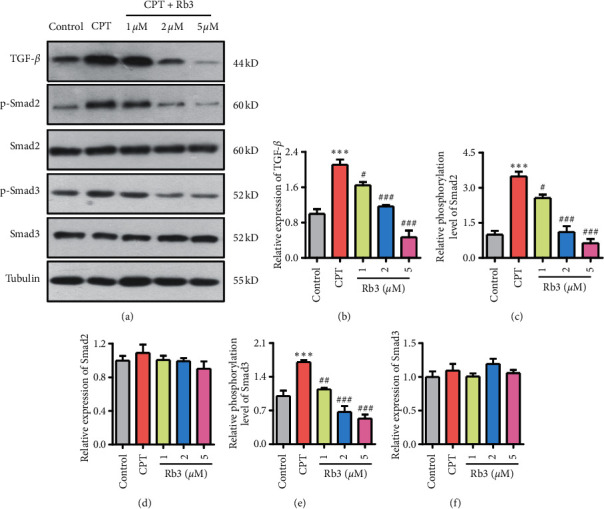
Rb3 regulated the TGF-*β* signal pathway in GP-293 cells. (a) Western blotting for the expression of TGF-*β*, Smad2, and Smad3 and phosphorylation of Smad2 and Smad3. (b–e) Quantitative analysis of densitometry for TGF-*β* (b), p-Smad2 (c), Smad2 (d), p-Smad3 (e), and Smad3 (f). ^*∗∗*^*p* < 0.01 and ^*∗∗∗*^*p* < 0.001 compared to the control group, and ^#^*p* < 0.05, ^##^*p* < 0.01, and ^###^*p* < 0.001 compared to the CPT group.

To further evaluate the effect of Rb3 on the TGF-*β* signal pathway, we detected the expression and phosphorylation level of Smad2 and Smad3 using Western blotting assay in human renal epithelial GP-293 cells. As shown in Figure 3, the expression level of TGF-*β* of cells exposed to CTP was induced compared with that in the control group which is consistent with the data mice (^*∗∗∗*^*p* < 0.001, Figure 3(b)), meanwhile CTP activated the phosphorylation level of Smad2 (^*∗∗∗*^*p* < 0.001, Figure 3(c)) and Smad3 (^*∗∗∗*^*p* < 0.001, Figure 3(e)) and but no statistical changing in the expression of Smad2 and Smad3. Following the treatment with Rb3 at different concentrations, the TGF-*β* expression level and phosphorylation level of Smad2 and Smad3 in GP-293 cells were suppressed compared to the CPT group, and its suppression level was concentration-dependent. Together, the data in vivo/in vitro imply that CPT could mediate TGF-*β* expression and its pathways and Rb3 possesses the suppression abilities to the CPT-mediated TGF-*β* signal pathway.

### 3.4. The Effects of Rb3 on CPT-Evoked Cellular Apoptosis in GP-293 Cells

**Figure 4:**
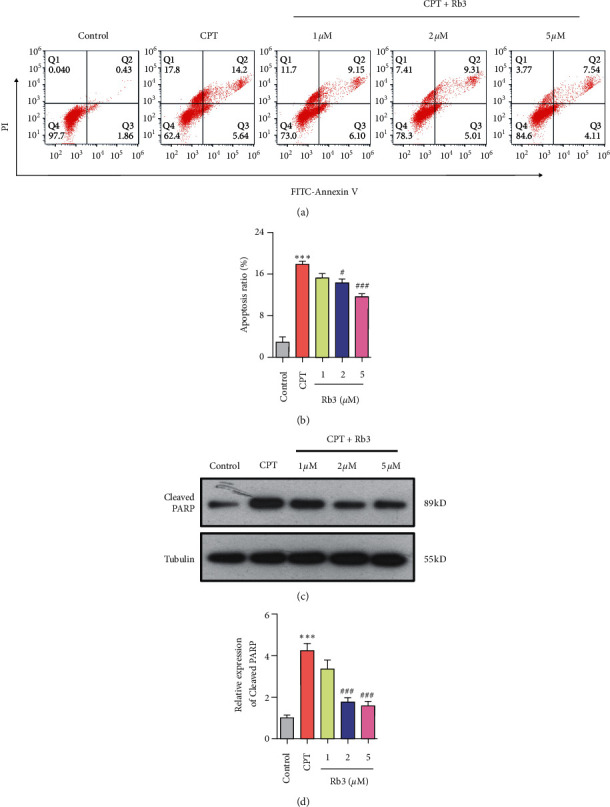
Rb3 suppressed CPT-evoked cellular apoptosis in GP-293 cells. (a) Flow cytometry of dual-stains of FITC-Annexin V and PI for cell apoptosis ratio. (b) Quantitative analysis for the apoptosis ratio by flow cytometry. (c) Western blotting for the cleavage level of PARP. (d) Quantitative analysis of densitometry for the cleaved PARP. ^*∗∗∗*^*p* < 0.001 compared to the control group; ^#^*p* < 0.05 and ^##^*p* < 0.01 compared to the CPT group.

To confirm the role of cellular apoptosis in Rb3-protecting CPT-evoked toxicity, flow cytometry of dual-staining of FITC-Annexin V/PI and Western blotting were performed to detect cellular apoptosis in human renal epithelial GP-293 cells. As shown in the quadrant scatter spot graphs of flow cytometry (Figure 4(a)), the cells in the control group exhibited an apoptosis ratio of only 2.88% ± 1.43%, while the apoptosis ratio of the cells exposed to CPT rose to 18.10% ± 1.28% (Figure 4(b)) and demonstrated that CPT significantly induced the cellular apoptosis in GP-293 cells. After the supplement of Rb3 at different concentrations, the apoptosis ratio of cells gradually decreased with the rising of Rb3 concentration, and the higher dose of 2 *μ*M and 5 *μ*M Rb3 had statistical differences in apoptosis ratio compared with that of the CPT group (^#^*p* < 0.05 in 2 *μ*M Rb3 and ^###^*p* < 0.001 in 5 *μ*M Rb3, Figures 4(a) and 4(b)). Furthermore, the cleaved level of PARP (poly ADP-ribose polymerase), a usual and classic indicator of apoptosis, was detected using Western blotting and displayed a consistent with the results of flow cytometry. The cleaved level of PARP in the CPT group was induced compared with that in the control group (^*∗∗∗*^*p* < 0.001), whereas Rb3 reversed the induction of the cleaved PARP level by CPT, and the higher dose is statistically significant differences (^###^*p* < 0.001 in 2 *μ*M and 5 *μ*m Rb3 compared to the CPT group, Figures 4(c) and 4(d)). All of these apoptosis results indicated that 2 *μ*M Rb3 just had initiated the suppression of the apoptosis induced by CPT in human renal epithelial GP-293 cells. Therefore, we choose the concentration of 2 *μ*M Rb3 for the follow-up experiments.

### 3.5. The Effects of Rb3 on CPT-Evoked Mitochondrial Apoptosis in GP-293 Cells

**Figure 5:**
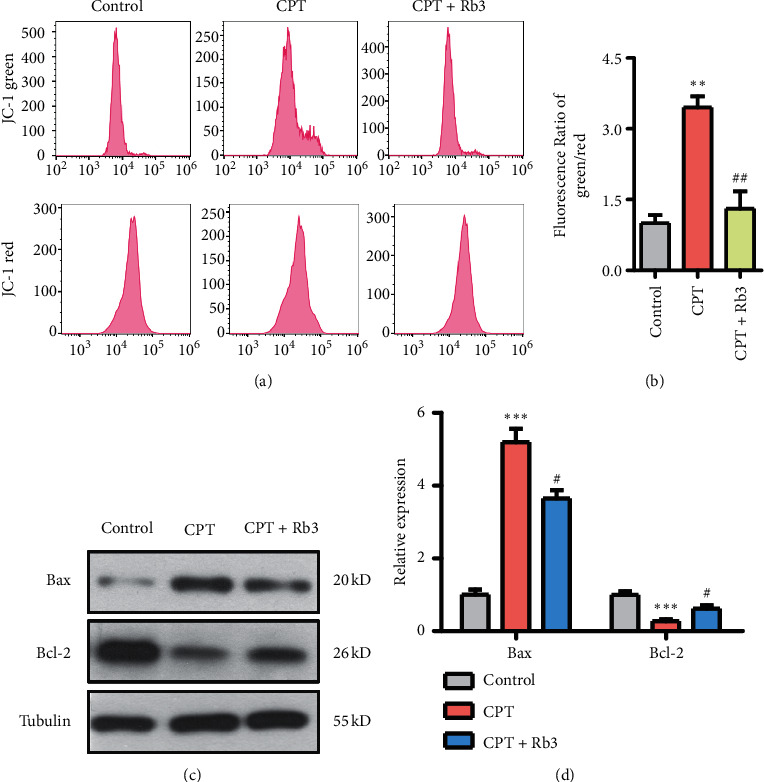
Rb3 suppressed CPT-evoked mitochondrial apoptosis in GP-293 cells. (a) Flow cytometry of JC-1 staining for mitochondrial membrane potential change. (b) Quantitative analysis for mitochondrial membrane potential change. (c) Western blotting for the expression levels of Bax and Bcl-2. (d) Quantitative analysis of densitometry for the expression of Bax and Bcl-2. ^*∗∗*^*p* < 0.01 and ^*∗∗∗*^*p* < 0.001 compared to the control group; ^#^*p* < 0.05 and ^##^*p* < 0.01 compared to the CPT group.

In order to determine whether mitochondrial participated in Rb3 suppressing cellular apoptosis evoked by CPT, the mitochondrial membrane potential of GP-293 cells was detected by flow cytometry of JC-1 staining, and mitochondrial-related protein expression of Bax and Bcl-2 was probed by Western blotting. The histogram from flow cytometry analysis (Figures 5(a) and 5(b)) showed that the fluorescence ratio of green/red in the cells of the CPT group is nearly four-folds to that in the control group and demonstrated that CPT induced mitochondrial depolarization which is the early event of cellular apoptosis. Reservedly, the administration of Rb3 at 2 *μ*M significantly repaired the mitochondrial depolarization induced by CPT, and its fluorescence ratio of green/red is similar to that in the control group. Additionally, Western blot showed that CPT induced the expression of Bax and inhibited Bcl-2 expression; meanwhile, Rb3 relieved the CPT-mediated protein expression of Bax and Bcl-2 in GP-293 cells (Figures 5(c) and 5(d)). Together, these results demonstrated that Rb3 might suppress the CPT-induced apoptosis via mediating mitochondrial depolarization and protein expression of Bcl-2 and Bax in GP-293 cells.

### 3.6. The Effects of Rb3 on Caspase 3, Caspase 8, and Caspase 9 in GP-293 Cells

**Figure 6:**
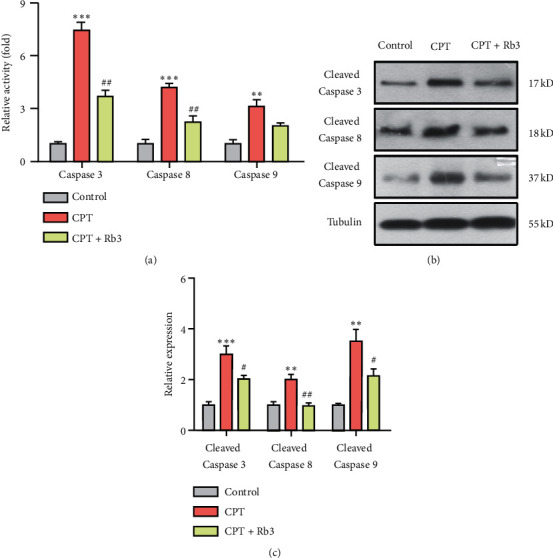
Rb3 regulated caspase 3, caspase 8, and caspase 9 in GP-293 cells. (a) Elisa assay for the activities of caspase 3, caspase 8, and caspase 9. (b) Western blotting for the cleavage levels of caspase 3, caspase 8, and caspase 9. (c) Quantitative analysis of densitometry for the cleavage levels of caspase 3, caspase 8, and caspase 9. ^*∗∗*^*p* < 0.01 and ^*∗∗∗*^*p* < 0.001 compared to the control group; ^#^*p* < 0.05 and ^##^*p* < 0.01 compared to the CPT group.

Caspases play an essential role in initiating and executing the cellular apoptosis process. The ELISA assay and Western blotting were employed to examine the activities and cleaved level of caspase 3, caspase 8, and caspase 9 in GP-293 cells, and the results are shown in Figure 6.  Figure 6(a) displayed that the cells exposed to CPT had an apparent increase of activities in caspase 3 (7.4 folds, ^*∗∗∗*^*p* < 0.001), caspase 8 (4.2 folds, ^*∗∗∗*^*p* < 0.001), and caspase 9 (3.1 folds, ^*∗∗*^*p* < 0.01) compared with those of cells in the control group, whereas Rb3 inhibited the activities of the three caspases induced by CPT in the varying degree. Besides, Western blotting showed that CPT induced the cleaved levels of caspases and Rb3 suppressed the induction (Figures 6(b) and 6(c)). The changing trends of the cleaved level of caspase 3, caspase 8, and caspase 9 are consistent with that of caspases activities in Figure 6(a).

### 3.7. The Roles of the TGF-*β* Signal Pathway on Rb3 Suppressing Endogenous Apoptosis Evoked by CPT in GP-293 Cells

**Figure 7:**
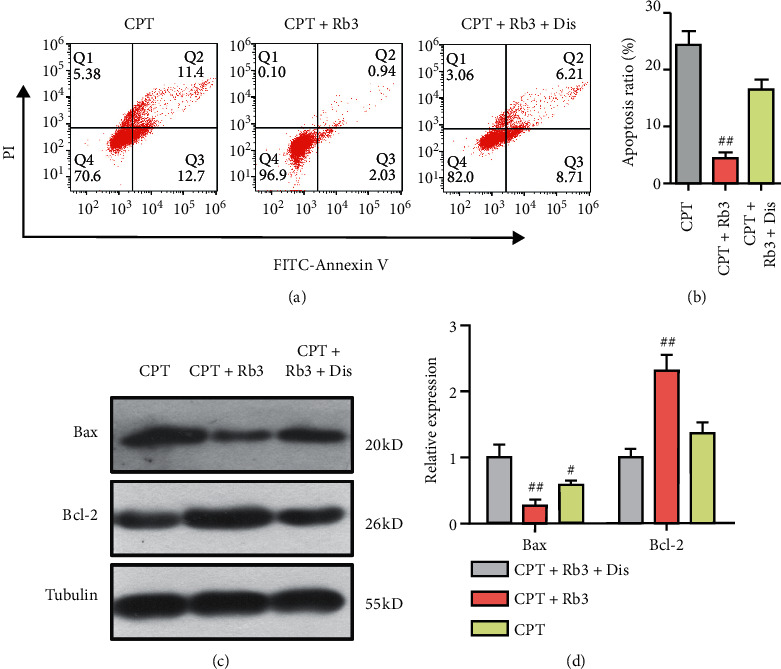
TGF-*β* signal pathway was essential to that Rb3 suppressed mitochondrial apoptosis evoked by CPT in GP-293 cells. (a) Flow cytometry of dual-stains of FITC-Annexin V and PI for cell apoptosis ratio. (b) Quantitative analysis for the apoptosis ratio by flow cytometry. (c) Western blotting for the expression level of Bax and Bcl-2. (d) Quantitative analysis of densitometry for the expression of Bax and Bcl-2. Dis, disitertide, an inhibitor of the TGF-*β* signal pathway. ^#^*p* < 0.05 and ^##^*p* < 0.01 compared to the CPT group.

The information mentioned above has shown that Rb3 could mediate the TGF-*β* signal pathway and endogenous apoptosis. To illuminate whether the TGF-*β* signal pathway is essential to Rb3 suppressing endogenous apoptosis evoked by CPT, we further used the TGF-*β* pathway inhibitor of disitertide in human renal epithelial GP-293 cells. As shown in Figure 7(a), the apoptosis ratio of the cells under the condition of CPT treatment was 24.3 ± 4.1% and Rb3 administration significantly suppressed the cellular apoptosis ratio to 4.3 ± 1.9%. However, after pretreatment with disitertide and Rb3, the cellular apoptosis ratio of 18.0 ± 3.1% was not a statistical decrease compared with that in the CPT group, which was opposite to that only Rb3 administration could just relieve the apoptosis ratio evoked by CPT. Moreover, Western blotting assay showed that Rb3 inhibited the expression of Bax and induced Bcl-2 expression compared with the CPT group, while combinational administration of Rb3 and disitertide deprived Rb3 of the rights mediating Bax and Bcl-2 expression. These results demonstrated that the TGF-*β* signal pathway is essential to Rb3 suppressing endogenous apoptosis evoked by CPT.

## 4. Discussion

CPT has been commonly used in the treatment of oral cancer clinically for several decades; however, the toxicity to normal tissue including the kidney limited its benefits as an effective antitumor agent. Although it was in the vague understanding that how CPT induced kidney toxicity, some shreds of evidence have shown that the pathway of NF-*κ*B, autophagy, oxidative stress, and apoptosis are associated with the kidney toxicity by CPT. Additionally, the TGF-*β* pathway plays a vital role in the kidney injury via kidney fibrosis, macrophage infiltration, and cellular apoptosis. However, the role of TGF-*β* in CPT-evoked kidney toxicity is rarely reported. In the present study, we found that CPT could induce the expression of TGF-*β* in the kidney tissue of the xenograft nude mice model bearing oral tumor and in the human renal epithelial cells GP-293; furthermore, CPT could also mediate the TGF-*β* signal pathway characterizing by the expression and phosphorylation of Smad2 and Smad3 in GP-293 cells. The results demonstrated that TGF-*β* might take part in CPT-evoked kidney toxicity.

Rb3 is a representative triterpenoid saponin isolated from ginseng, which could exert various biological activities in the apoptosis, oxidative stress, inflammation, and others. Recent studies suggested that Rb3 could serve as a protective agent to alleviate the injury of kidney tissue, cardiomyocyte, and hippocampus [27, 28]. The study by Xing et al. revealed that Rb3 protected against the CPT-induced kidney toxicity via the autophagy pathway mediated by AMPK and apoptosis [17]. In another study, Rb3 was reported to exert the cardioprotective effects via the activation of the antioxidation signaling pathway of PERK/Nrf2/HMOX1 in vivo and vitro [29]. In the current research, we illustrated a novel mechanism that Rb3 might protect against CPT-evoked kidney toxicity via the TGF-*β* signal pathway in vivo and in vitro. The mechanism confirmed that Rb3 suppressed the expression of TGF-*β* induced by CPT in the kidney tissue of the xenograft nude mice model bearing oral tumor and in renal cells GP-293 and phosphorylation of Smad2 and Smad3 in GP-293 cells.

Apoptosis is a physiologically programmed cell death process to get rid of the unwanted and dysfunctional cells for maintaining the homeostasis of the body [30]. Mitochondrial, recognized as the regulated center of cellular activities, controls the respiratory chain and oxidative phosphorylation serving for nearly all of the cellular physiology. In the apoptosis progression, mitochondrial depolarization occurs as response to apoptosis stimuli, cytochrome C releases from mitochondrial, and the expression of apoptosis-related factor Bcl-2 and Bax in mitochondrial changes; following actives, the series of cysteinyl aspartate specific proteinase (caspase) of caspase 3, caspase 8, and caspase 9 eventually induced the cleavage of poly ADP-ribose polymerase (PARP) and cell death [31]. Any disordered mitochondrial apoptosis section would cause functional cell death and trigger the normal tissue injury [32]. The research studies had confirmed that CPT evoked the kidney cell apoptosis via inducing Bax expression, inhibiting Bcl-2 expression, and activating caspase activities. TGF-*β* was also reported to take part in the cell apoptosis via mitochondrial. Our results implied that TGF-*β* partly contributed to the CPT-induced mitochondrial apoptosis. Moreover, the present study revealed that TGF-*β* is essential to Rb3 protecting against CPT-evoked apoptosis, which confirmed that Rb3 lost the protective effect in the pretreatment with the TGF-*β* inhibitor of disitertide in kidney cells.

## 5. Conclusions

In conclusion, the TGF-*β* pathway might contribute to CPT-evoked kidney toxicity, and Rb3 could exert a protective effect on the kidney toxicity through the TGF-*β* pathway-mediated mitochondrial apoptosis during the treatment of oral cancer by CPT. Our finding provides a novel insight into the protective effect of Rb3 on CPT-evoked kidney toxicity. It is very significant for the development of Rb3 and the treatment of oral cancer in the clinic.

## Data Availability

The data generated or analyzed to support the findings of this study are included within the article.

## References

[1] Ghantous Y., Abu Elnaaj I. (2017). Global incidence and risk factors of oral cancer. *Harefuah*.

[2] D’Souza S., Addepalli V. (2018). Preventive measures in oral cancer: an overview. *Biomedicine & Pharmacotherapy = Biomedecine & Pharmacotherapie*.

[3] Shibahara T. (2017). Oral cancer diagnosis and therapy. *Clinical Calcium*.

[4] Keshavarzi M., Darijani M., Momeni F. (2017). Molecular imaging and oral cancer diagnosis and therapy. *Journal of Cellular Biochemistry*.

[5] Varela-Centelles P., Castelo-Baz P., Seoane-Romero J. (2017). Oral cancer: early/delayed diagnosis. *British Dental Journal*.

[6] Kanagalingam J., Wahid M. I. A., Lin J.-C. (2018). Patient and oncologist perceptions regarding symptoms and impact on quality-of-life of oral mucositis in cancer treatment: results from the awareness drives oral mucositis perception (ADOPT) study. *Supportive Care in Cancer*.

[7] Valdez J. A., Brennan M. T. (2018). Impact of oral cancer on quality of life. *Dental Clinics of North America*.

[8] Zhou J., Yu G., Huang F. (2017). Supramolecular chemotherapy based on host-guest molecular recognition: a novel strategy in the battle against cancer with a bright future. *Chemical Society Reviews*.

[9] Kirita T. (2014). Oral cancer: current status of molecular biology and treatment strategy. *International Journal of Clinical Oncology*.

[10] Hartner L. (2018). Chemotherapy for oral cancer. *Dental Clinics of North America*.

[11] Hsieh M.-T., Huang L.-J., Wu T.-S. (2018). Synthesis and antitumor activity of bis(hydroxymethyl)propionate analogs of pterostilbene in cisplatin-resistant human oral cancer cells. *Bioorganic & Medicinal Chemistry*.

[12] Sato K., Hayashi Y., Watanabe K., Yoshimi R., Hibi H. (2019). Concurrent chemoradiotherapy with intravenous cisplatin and docetaxel for advanced oral cancer. *Nagoya Journal of Medical Science*.

[13] Nguyen-Tan P. F., Zhang Q., Ang K. K. (2014). Randomized phase III trial to test accelerated versus standard fractionation in combination with concurrent cisplatin for head and neck carcinomas in the radiation therapy oncology group 0129 trial: long-term report of efficacy and toxicity. *Journal of Clinical Oncology*.

[14] Anderson C. M., Lee C. M., Saunders D. P. (2019). Phase IIb, randomized, double-blind trial of GC4419 versus placebo to reduce severe oral mucositis due to concurrent radiotherapy and cisplatin for head and neck cancer. *Journal of Clinical Oncology*.

[15] Adams C. J., Grainger M. N. C., Manley-Harris M. (2015). Isolation of maltol glucoside from the floral nectar of New Zealand mānuka (*Leptospermum scoparium*). *Food Chemistry*.

[16] Xing J. J. (2019). Supplementation of saponins from leaves of panax quinquefolius mitigates cisplatin-evoked cardiotoxicity via inhibiting oxidative stress-associated inflammation and apoptosis in mice. *Antioxidants (Basel)*.

[17] Xing J. J. (2019). Ginsenoside Rb3 provides protective effects against cisplatin-induced nephrotoxicity via regulation of AMPK-/mTOR-mediated autophagy and inhibition of apoptosis in vitro and in vivo. *Cell Prolif*.

[18] Schmierer B., Hill C. S. (2007). TGF*β*-SMAD signal transduction: molecular specificity and functional flexibility. *Nature Reviews Molecular Cell Biology*.

[19] Pines M. (2008). Targeting TGF*β* signaling to inhibit fibroblast activation as a therapy for fibrosis and cancer: effect of halofuginone. *Expert Opinion on Drug Discovery*.

[20] Iyer S., Wang Z.-G., Akhtari M., Zhao W., Seth P. (2005). Targeting TGF beta signaling for cancer therapy. *Cancer Biology & Therapy*.

[21] Xu T.-B., Li L., Luo X.-D., Lin H. (2017). BMSCs protect against liver injury via suppressing hepatocyte apoptosis and activating TGF-*β*1/Bax singling pathway. *Biomedicine & Pharmacotherapy*.

[22] Fan X.-D., Zheng H.-B., Fan X.-S., Lu S. (2018). Increase of SOX9 promotes hepatic ischemia/reperfusion (IR) injury by activating TGF-*β*1. *Biochemical and Biophysical Research Communications*.

[23] Jin J., Sun H., Liu D. (2019). LRG1 promotes apoptosis and autophagy through the TGF*β*-smad1/5 signaling pathway to exacerbate ischemia/reperfusion injury. *Neuroscience*.

[24] Jiang X., Xiang G., Wang Y. (2012). MicroRNA-590-5p regulates proliferation and invasion in human hepatocellular carcinoma cells by targeting TGF-*β* RII. *Molecules and Cells*.

[25] Jeong S.-Y., Seol D.-W. (2008). The role of mitochondria in apoptosis. *BMB Reports*.

[26] Tusskorn O., Khunluck T., Prawan A., Senggunprai L., Kukongviriyapan V. (2019). Mitochondrial division inhibitor-1 potentiates cisplatin-induced apoptosis via the mitochondrial death pathway in cholangiocarcinoma cells. *Biomedicine & Pharmacotherapy*.

[27] Ma L. (2014). Ginsenoside Rb3 protects cardiomyocytes against ischemia-reperfusion injury via the inhibition of JNK-mediated NF-kappaB pathway: a mouse cardiomyocyte model. *PLoS ONE*.

[28] Wang T. (2010). Effect of ginsenoside Rb3 on myocardial injury and heart function impairment induced by isoproterenol in rats. *European Journal of Pharmacology*.

[29] Sun J., Yu X., Huangpu H., Yao F. (2019). Ginsenoside Rb3 protects cardiomyocytes against hypoxia/reoxygenation injury via activating the antioxidation signaling pathway of PERK/Nrf2/HMOX1. *Biomedicine & Pharmacotherapy*.

[30] Elmore S. (2007). Apoptosis: a review of programmed cell death. *Toxicologic Pathology*.

[31] Lopez J., Tait S. W. G. (2015). Mitochondrial apoptosis: killing cancer using the enemy within. *British Journal of Cancer*.

[32] Schapira A. H. (2012). Mitochondrial diseases. *The Lancet*.

